# A state-wide population-based program for detection of Lynch syndrome based upon immunohistochemical and molecular testing of colorectal tumours

**DOI:** 10.1186/1897-4287-10-S2-A18

**Published:** 2012-04-12

**Authors:** L Schofield, F Grieu, J Goldblatt, B Amanuel, B Iacopetta

**Affiliations:** 1 Genetic Services of Western Australia, King Edward Memorial Hospital, Subiaco, Australia; 2 School of Surgery, University of Western Australia, Nedlands, Australia; 3 School of Paediatrics and Child Health, University of Western Australia, Nedlands, Australia; 4 Molecular Pathology, PathWest, Nedlands, Australia

## Background

We have previously established in a large retrospective study that testing for microsatellite instability (MSI) in colorectal cancer (CRC) from patients aged <60 years was an effective first screen to identify individuals with Lynch syndrome (LS). From these findings, MSI and/or immunohistochemical (IHC) screening was recommended for all newly diagnosed CRC patients aged <60 years in Western Australia, regardless of family history of cancer. In the current study we evaluated the utility of routine MSI/IHC screening by diagnostic pathology laboratories for the detection of previously undiagnosed individuals and families with LS.

From January 2009 to December 2010, 270 tumours were tested for MSI and for expression of MLH1, PMS2, MSH2 and MSH6 using IHC. Cases showing MSI and/or loss of expression were also tested for the *BRAF* V600E hotspot mutation. Seventy cases were found to have MSI, of which 25 were excluded from further investigation as possible LS cases due to presence of the *BRAF* V600E mutation. The remaining 45 “red flag” cases were eligible for germline testing based on their MSI, IHC and *BRAF* status. From 26 cases tested to date, 11 germline mutations have been found. Nine were from individuals not previously recognized as LS and two were untested members from known LS families. Extrapolation of the mutation incidence (11/26, 42%) to all red flag cases (n=45) suggests that approximately 19 mutation carriers exist in this cohort. This value approximates the number of LS cases that could be expected to arise in the Western Australian population over a two-year period (n=24), assuming that 1% of all CRCs are due to LS.

Although further improvements in workflow can be made, our preliminary findings following the implementation of state-wide routine MSI and IHC testing in Western Australia indicate that the majority of LS cases are being identified.

